# Novel 3D-Printed Biocarriers from Aluminosilicate Materials

**DOI:** 10.3390/ma16134826

**Published:** 2023-07-05

**Authors:** Eleni Anna Economou, Savvas Koltsakidis, Ioanna Dalla, Konstantinos Tsongas, George Em. Romanos, Dimitrios Tzetzis, Polycarpos Falaras, George Theodorakopoulos, Vesna Middelkoop, Themistoklis Sfetsas

**Affiliations:** 1 QLAB Private Company, Research and Development, Quality Control and Testing Services, 57008 Thessaloniki, Greece; ea.oikonomou@q-lab.gr (E.A.E.);; 2 Digital Manufacturing and Materials Characterization Laboratory, School of Science and Technology, International Hellenic University, 57001 Thessaloniki, Greece; 3 Department of Industrial Engineering and Management, School of Engineering, International Hellenic University, 57001 Thessaloniki, Greece; 4 Institute of Nanoscience and Nanotechnology, National Center of Scientific Research “Demokritos”, Agia Paraskevi, 15310 Athens, Greece; 5 Sustainable Materials Management, Flemish Institute for Technological Research, VITO (Vlaamse Instelling voor Technologisch Onderzoek), Boeretang 200, 2400 Mol, Belgium

**Keywords:** aluminosilicate clays, zeolites, biocarriers, inorganic nanotubes, 3D printing

## Abstract

The addition of biocarriers can improve biological processes in bioreactors, since their surface allows for the immobilization, attachment, protection, and growth of microorganisms. In addition, the development of a biofilm layer allows for the colonization of microorganisms in the biocarriers. The structure, composition, and roughness of the biocarriers’ surface are crucial factors that affect the development of the biofilm. In the current work, the aluminosilicate zeolites 13X and ZSM-5 were examined as the main building components of the biocarrier scaffolds, using bentonite, montmorillonite, and halloysite nanotubes as inorganic binders in various combinations. We utilized 3D printing to form pastes into monoliths that underwent heat treatment. The 3D-printed biocarriers were subjected to a mechanical analysis, including density, compression, and nanoindentation tests. Furthermore, the 3D-printed biocarriers were morphologically and structurally characterized using nitrogen adsorption at 77 K (LN_2_), scanning electron microscopy (SEM), and X-ray diffraction (XRD). The stress–strain response of the materials was obtained through nanoindentation tests combined with the finite element analysis (FEA). These tests were also utilized to simulate the lattice geometries under compression loading conditions to investigate their deformation and stress distribution in relation to experimental compression testing. The results indicated that the 3D-printed biocarrier of 13X/halloysite nanotubes was endowed with a high specific surface area of 711 m^2^/g and extended mesoporous structure. Due to these assets, its bulk density of 1.67 g/cm^3^ was one of the lowest observed amongst the biocarriers derived from the various combinations of materials. The biocarriers based on the 13X zeolite exhibited the highest mechanical stability and appropriate morphological features. The 13X/halloysite nanotubes scaffold exhibited a hardness value of 45.64 MPa, which is moderate compared to the rest, while it presented the highest value of modulus of elasticity. In conclusion, aluminosilicate zeolites and their combinations with clays and inorganic nanotubes provide 3D-printed biocarriers with various textural and structural properties, which can be utilized to improve biological processes, while the most favorable characteristics are observed when utilizing the combination of 13X/halloysite nanotubes.

## 1. Introduction

Zeolites are highly porous crystalline aluminosilicates formed by SiO_4_ and AlO_4_ tetrahedra. These materials have a wide range of applications such as sorbents, ion exchangers in detergents, and catalysts in the oil refining and petrochemical industry and in waste treatment processes [1,2,3]. 

In bioreactors, during waste treatment, a variety of byproducts might be produced depending on the digestion type performed. The application of biocarriers is one way of increasing productivity in bioreactors. Biocarriers frequently consist of porous surfaces where bacteria can adhere to create biofilms. They can be manufactured from reactive organic materials (alginate), inert materials (polyvinyl alcohol or PVA), or inorganic materials (zeolite). Due to the increased surface area, biocarriers increase the productivity of the bioreactor and shield the attached microorganisms from the forces of the bioreactor [4].

Various methods are available for the synthesis of zeolite monolithic structures such as extrusion [5], inverse replication [6,7], dry gel conversion [8], 3D printing [9,10,11], wash coating [12], hydrothermal coating [13] or a combination of the last two [14]. However, not all of these can be applied in the synthesis of bulk zeolite structures, as the final material needs to have high porosity for the accessibility of the pores and active sites. Some methods of zeolite 3D printing, such as direct ink writing (DIW), are similar to the less sophisticated method of extrusion, which has already been widely investigated [5]. However, the impact of different binders for manufacturing zeolite monoliths through 3D printing for wastewater treatment is not yet fully understood.

The applications of 3D printing for manufacturing functional components for chemical, biochemical and environmental processes have significantly increased in recent years [15]. The 3D printing technologies have an advantage over conventional production techniques by allowing for the bespoke design of the biocarrier, achieving customized shape, microstructure, and multi-material compositions for homogeneous dispersion of the active species, as well as efficient diffusion and mass transfer. In addition, the application of 3D printing technology has various benefits, including less waste produced during geometry machining and reduced material consumption for geometry fabrication [16]. The type of materials utilized to create the ceramic biocarriers has a significant impact on both the porosity and mechanical strength of the printed structure. In contrast to metals and polymers, ceramics—which are mixtures of metallic and nonmetallic elements—are typically insulative to the passage of electricity and heat and more resilient to high temperatures and harsh environments [17]. In ceramics, binder materials are used to increase the mechanical strength of ceramic bodies so they can withstand the production processes before calcination without breaking. In order to increase surface adherence and slow drying, binders are glues that solidify ceramic powders as they dry [18]. Some recent studies have highlighted the effect of binder on the physicochemical properties of zeolite monoliths. Researchers in [19] created monoliths using ZSM-5 and examined how the concentration of bentonite clay binder impacts the rheological, physicochemical, and mechanical characteristics. Their findings revealed that a higher concentration of binder leads to a more uniform material with enhanced mechanical strength but deteriorates the physicochemical properties. In another study [20], researchers found that the final sample’s porosity is decreased by the frequent use of inorganic binders at high concentrations. In [21], research investigations demonstrated that the choice of binder in the formulation of 3D-printed structures significantly influences the printing process, the calcination stage, and the physicochemical properties of the resulting structure.

In this work, two zeolites were selected as the basic structural components. The selection was made primarily due to their different Si/Al ratios, which ranged from extremely low (13X) to high (ZSM-5), but also due to the variation of their pore structural features (pore size and shape, tortuosity, porosity) and the large difference of their particle size (0.5 μm for ZSM-5 and 5 μm for 13X). The inorganic binders utilized are inorganic halloysite nanotubes, montmorillonite, and bentonite. It was established which zeolite/binder combinations, and in what mass ratio, were the most suitable for developing biocarriers with enhanced resistance to attrition and erosion and improved structural and morphological features to augment the bioprocess performance [22]. This was determined by carrying out comprehensive formulation screening with the different Si/Al ratios of the selected zeolites combined with the silicon-rich structure and the different size, shape, and expanding capacity of the above-mentioned phyllosilicate. The 3D printing technology is used to develop carrier aluminosilicate scaffolds to efficiently immobilize enzymes for tailored continuous flow biocatalytic reactors. The resulting 3D-printed biocarriers underwent mechanical testing to evaluate their density, compression, and nanoindentation. Additionally, the biocarriers were analyzed using techniques such as nitrogen adsorption at 77 K (LN2), scanning electron microscopy (SEM), energy-dispersive X-ray spectroscopy (EDX), and X-ray diffraction (XRD) to examine their structural and morphological characteristics.

## 2. Materials and Methods

Ceramic material ZSM-5 (Thermo Fisher Scientific Inc., Waltham, MA, USA) or 13X zeolite (Alfa Aesar, Ward Hill, MA, USA) was combined with inorganic phyllomorphous porous solids (montmorillonite or bentonite, or halloysite nanotubes) and an organic binder to create the printing paste. Subsequently, a colloidal silicon/ionized aqueous solution was created. For the effective production of the printed ceramic paste, the paste’s solid content must be more than 50% in order to obtain maximum density and avoid excessive shrinkage during drying and calcination at 500 °C. Finally, the colloidal silicon/ionized water mixture was gradually blended with the solid mixture. The final mixture was mechanically agitated until it took on the consistency of a viscous paste. To decrease agglomeration that occurred during preparation, the paste was transferred to an iron plate and handled with a metal spatula. For the complete subtraction of aggregates, a sieve with a diameter of 45 μm was utilized. A metal spatula was used to push the paste through the pores of the sieve. The pore diameter of the final agglomerate-free paste was less than 45 μm.

**Figure 1:**
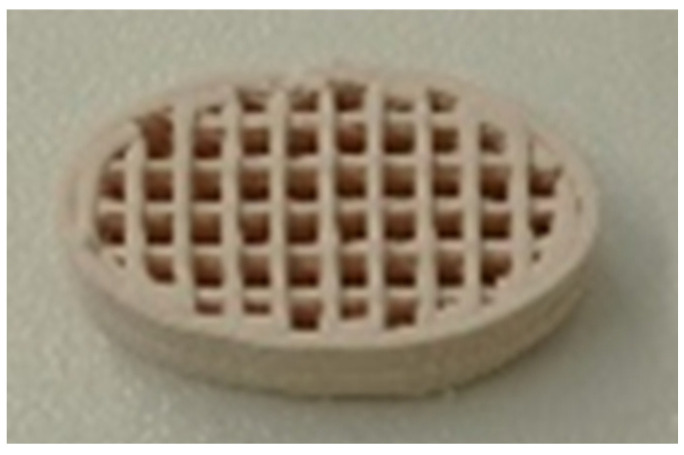
Novel 3D-printed zeolite biocarrier.

A Cellink’s Bio X6 (Gothenburg, Sweden) printer, which is based on direct ink writing (DIW), was utilized to print the biocarriers. Following the manufacturing process, the paste was carefully inserted into the syringe to avoid the formation of air bubbles. The material was then centrifuged to eliminate any remaining air bubbles. The syringe was then inserted into the printer, where print preparation occurred. The 3D geometry was created, and parameters were set, such as the shape of the biocarriers, the recurring geometric pattern, the height of each layer, the nozzle diameter, the pressure, and the printing speed set. The chosen geometric pattern can be defined as rectilinear. Each layer was printed in a single direction, with the next layer printed perpendicular to the previous one (90°). Its dimensions were 24 mm wide, 14 mm length, and 7 mm height. The density of the repeating geometric pattern was 18% (Figure 1).

For high-resolution printing attachments, the particles must be at least around ten times smaller than the diameter of the nozzle used to print the paste, ensuring that the mixture is homogenous and free of agglomerations. Table 1 shows the percentage of ceramic material and inorganic binder content in the biocarriers’ printing paste. 

The zeolite and alumina silicate binders were made in various quantities, with solids contents ranging from 44% to 61%. Due to the clarity level being kept low, the final structure was primarily zeolite while retaining good mechanical strength. The ratio of colloidal silicon to clay was likewise kept constant at 2.5. The zeolite and argil mixing ratio in the paste was 8:1, with relative amounts of 89% and 11%, respectively. The Bio X6 printer is capable of printing a volume of 128 mm × 90 mm × 90 mm (W × L × H) with an accuracy of 1 μm (arm movement). Furthermore, the printer allows print heads to be heated from 4 °C to 250 °C, allowing the viscosity of ceramic pastes to be adjusted, while the print surface can be cooled to 4 °C for better-printed structures. 

An Autosorb-1-MP adsorption analyzer of Quantachrome was used to obtain the N_2_ adsorption–desorption isotherms (77 K) and extract the pore structural properties including the BET surface, the micropore and mesopore volume and the pore size distributions. For the latter, the non-local density functional theory (NLDFT) method for silica and N_2_ as adsorbate was applied to interpret both the adsorption (adsorption model cylindrical pores) and desorption (equilibrium model cylindrical pores) branches of the isotherm. Before the measurement, all samples were outgassed at high temperature (300 °C) and high vacuum conditions (10^−5^ mbar) for 24 h. Macroscopic pieces (3 × 3 mm) of the 3D-printed biocarriers were placed into large bulb sample cells with stem ID of 7 mm. The pieces were cut with a diamond disk. This way we avoided haphazard breaking and milling of the biocarriers that could affect their microstructure.

A Netzsch STA 449 C Jupiter was employed to measure the thermogravimetric (TG) and derivative thermogravimetric (DTG) curves. A Jeol JSM-7401F field emission scanning electron microscope equipped with Gentle Beam mode was employed to characterize the surface morphology of the biocarriers. Gentle Beam technology can reduce charging and improve resolution, signal-to-noise ratio, and beam brightness. Scanning electron microscopy (SEM) was performed at typical conditions of 10 mA of emission current and 2 kV of operating voltage. Additional SEM images and elemental mappings were obtained using a FEGFEI Nova NanoSEM 450 instrument operating at an accelerating voltage of 20 keV equipped with an energy dispersive X-ray (EDX) spectroscopy Bruker QUANTAX 200 system.

The XRD diffraction patterns were recorded on a Rigaku R-AXIS IV imaging plate detector mounted on a Rigaku RU-H3R rotating anode X-ray generator (operating at 50 kV, 100 mA, nickel-filtered Cu Ka1 radiation). The samples were sealed in Lindemann capillaries. The X-ray diffraction (XRD) technique is used to study the parameters of material crystallinity. Further XRD analysis was performed using a Panalytical Empyrean operating at a voltage of 45 kV and a current of 40 mA.

The greatest compressive stress that a given solid material can withstand without breaking is known as compressive strength [23]. To determine the compressive strength of the biocarriers that were printed and thermally treated, uniaxial tests were conducted on the samples. The tests were carried out at room temperature using a universal testing machine (M550-50AT, Testometric, Rochdale, UK) with a crosshead that moved at a constant rate of 1 mm/min. The loads applied during the tests were recorded using a loadcell with a resolution of 1 mN. To calculate stress, the compressive force was divided by the measured cross-sectional area of each sample. A minimum of 3 samples were tested for each composition, and the compressive strength was determined by identifying the maximum stress reached on the force-displacement curves obtained from the tests.

To improve the accuracy of the compression tests for the biocarriers, a finite element model (FEM) has been developed [24]. The ANSYS™ software (Ansys^®^ Academic Research Mechanical, Release 23.1) was used to investigate the mechanical behavior of various structures. The explicit dynamic module was employed to accurately simulate the response of lattices with dynamic finite element analyses. This process helped to capture their deformations, nonlinear material behavior, and different fracture modes. A model that combines damage mechanics and plasticity was developed to analyze the failure of ceramic structures. The objective was to create a model that accurately describes the key aspects of the failure process when ceramics are subjected to uniaxial loading. To achieve this, an effective stress-based plasticity model is combined with a damage model that considers plastic and elastic strain measures. The model’s response under compression was compared to experimental data. To obtain mesh-independent results, mesh sensitivity analyses were conducted using convergence studies on normalized elastic modulus, as per the literature. The studies revealed that convergence was achieved with nearly 100,000 elements for each model. Hexahedral elements were utilized with a size of 0.1 to 0.5 mm (average 0.3 mm) to fit the complex geometry of lattice structures.

The nanoindentation measurements were carried out on a DUH-211S Shimadzu (Kyoto, Japan) device with a diamond triangular tip Berkovich indenter (angle of 65°, tip radius is 100 nm) and a force resolution of 0.196 μN. A material’s resistance to localized plastic deformation, such as a minor dent or scratch, is measured by its hardness [17]. The elastic modulus, also referred to as the modulus of elasticity, is a measurement of a material’s resistance to undergoing elastic deformation when under stress [25]. The modulus and hardness were determined based on the work of Oliver and Pharr [26,27,28]. The maximum load observed was 100 mN, and it was achieved with a rate of 6.662 mN/s.

## 3. Results

### 3.1. Pore Structural and Textural Features of the Novel 3D-Printed Biocarriers

**Figure 2:**
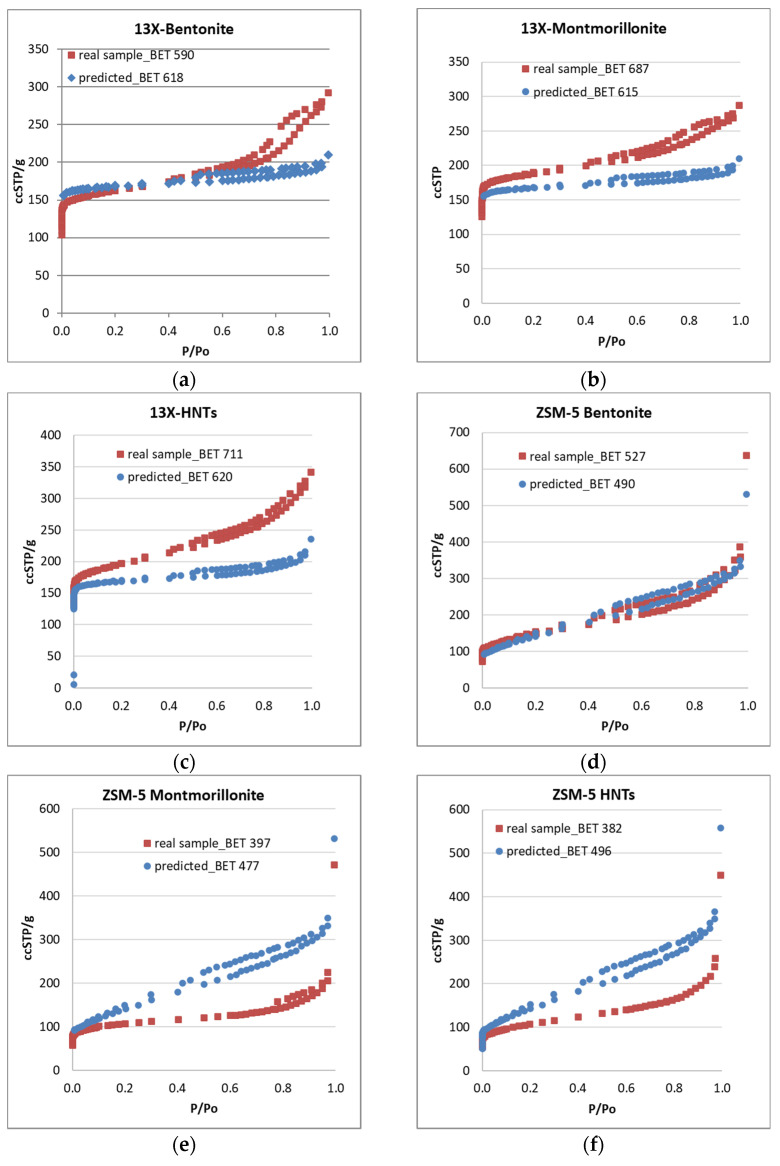
Comparison between the real and predicted N_2_ adsorption–desorption isotherms (77Κ) of the biocarriers examined in this work: (**a**–**c**) 13X with bentonite, montmorillonite, and HNTs; (**e**–**f**) ZSM-5 with bentonite, montmorillonite, and HNTs.

Nitrogen adsorption (77 K) isotherms were obtained not only for the shaped biocarriers but also for all the inorganic materials involved in their development. Apart from elaborating the pore structural and textural features of the biocarriers’ basic components, the main target of this approach was to achieve the prediction of the nitrogen adsorption isotherms of the end products, being aware of the mass ratio of their basic components (zeolites/inorganic binders, 8:1) and assuming that the final structure entails solely a physical mixing process of the two powders. The adsorption isotherm is predicted with the assumption that the use of inorganic and organic binders along with the further calcination of the raw biocarriers do not affect the pore structural characteristics of the parent zeolites. Thus, we admit that the N_2_ adsorption isotherm of the calcined structure coincides with the one corresponding to a sample produced by physically mixing the two powders (zeolite and inorganic binder) at a specific mass ratio. Nonetheless, significant deviations are expected between the predicted isotherm and that of the real sample. Negative deviations could be related to the loss of a fraction of the pore volume due to sintering or due to carbon remnants of the organic binder after calcination (blockage of the pore entrance). It is also possible that part of the meso-macropore structure of the parent zeolite is lost due to coverage by the binder particles in a core–shell structure. The more interesting cases, however, are those where synergistic effects arise due to the size, shape, surface chemistry, expanding capacity, and nesting conformation of the inorganic binder particles, which points to positive deviations of the real adsorption isotherm compared to the predicted one. As such, the interpretation of the N_2_ adsorption isotherms (77 K) of the real samples led to the pore structural properties presented in Table 2, while a comparison of the real and predicted isotherms of the shaped biocarriers is depicted in Figure 2a–f. 

The results included in Table 2 indicate that the 13X zeolite is an almost purely microporous material with a narrow pore size distribution centered around 10 Å. As such, 13X exhibited larger specific surface area, micropore volume, and micro- and mesopore size than ZSM-5 zeolite. The ZSM-5 zeolite, on the other hand, has more complex characteristics, possessing an hierarchical pore structure with distinctive bimodal pore size distribution and a higher total pore volume and mesopore volume than the 13X zeolite (Table 2).

The various zeolite/inorganic binder combinations result in the development of complex materials with novel properties and applications. The 13X/montmorillonite had a greater total pore volume and a bigger volume of mesopores than the pure 13X zeolite, but it also had a smaller specific surface area, as well as a smaller volume and diameter of micropores. The 13X/bentonite had larger total pores and mesopores volume than 13X but showed a decrease in specific surface area, micropore volume, and diameter.

Although the 13X zeolite had a high specific surface area, its combination with the inorganic binders reduced it, with the exception of 13X/halloysite nanotubes, which showed an increase in specific surface area, total volume of pores, and mesopore volume. However, the diameter of the micropores was smaller. The ZSM-5/bentonite mixture exhibited similar behavior, with a larger diameter value than pure ZSM-5.

The general characteristics of the composites containing 13X and ZSM-5 zeolites decreased as expected due to the lower specific surface area and micro- and mesopore volume of the inorganic binders. The best complex zeolites, according to BET analysis, were 13X/halloysite nanotubes and ZSM-5/bentonite. The same conclusion is drawn by examining the comparison plots of the real and predicted N_2_ adsorption isotherms (77 K) of the 3D-printed biocarriers (Figure 2a–f). It can be seen that in all cases where the 13X zeolite was used as the core material of the biocarrier (Figure 2a–c), the real isotherm of the 3D-printed scaffold unveils higher mesopore volume than the predicted one. In some cases, this trend is monotonic covering the entire range of pore sizes, from the micropores to the macropores. Only in the case of the 13X/bentonite sample, the real micropore volume was lower than the one derived from the predicted isotherm, and this may be connected with the expanding capacity of bentonite compared to the other two binders. In general, there is an impressive synergistic effect achieved by the mixing/3D-printing and calcination of the 13X zeolite with all the inorganic binders that leads to enhanced mesoporosity, a feature that was missing from the parent 13X zeolite material. In fact, the mesopore volume of the obtained biocarriers is larger than that of each individual component. On the contrary, in all cases that ZSM-5 was involved as the core material for the deployment of the biocarriers, the real isotherms show moderate pore structural features as compared to the ones derived from the predicted isotherm (Figure 2e,f). This different trend can be attributed to the variation in the morphology and size of the crystallites of the two zeolites and to their highly different surface acidity (Si:Al ratio) that may lead to better dispersion of the binder’s particles around the particles of the zeolitic material (13X), thus concluding to a more effective sintering process during the calcination of the raw biocarrier. Another cause can be related to the different pore structure of the two zeolites. ZSM-5 is characterized by smaller micropores and a quite extended mesopore structure as compared to 13X. As such, the mixing with the inorganic binder seems to have had an adverse effect on the mesopore structure of the ZSM-5 zeolite. This is also confirmed by the results presented in Table 2 showing that the shaped biocarrier scaffold exhibits lower mesopore volume compared to the pristine ZSM-5 material. 

**Figure 3:**
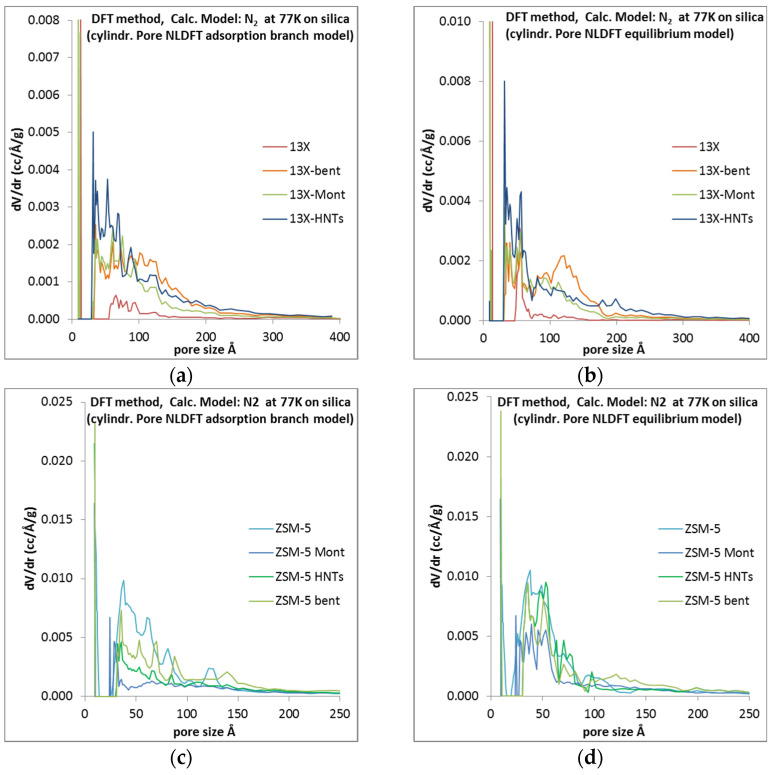
NLDFT-derived PSD of 13X, ZSM-5, and the respective biocarriers: (**a**) 13X and biocarriers adsorption branch; (**b**) 13X and biocarriers desorption branch; (**c**) ZSM-5 and biocarriers adsorption branch; (**d**) ZSM-5 and biocarriers desorption branch.

Figure 2 presents the NLDFT-derived pore size distribution (PSD) of all 3D-printed biocarriers in comparison to that of 13X and ZSM-5, showing the pore structural alterations of the parent zeolites due to their structuring in the form of biocarriers. Hence, in all cases where 13X was used as the core building material of the biocarrier, the addition of the inorganic binders and the shaping of the powder mixture into the form of a 3D-printed scaffold led to the creation of an extra mesoporous structure. Importantly, in all 13X-based scaffolds the mesopore structure has hierarchical characteristics, due to a significant number of pores with completely different sizes belonging to the mesopore range. Especially Figure 3a, the NLDFT-derived PSD of the adsorption branch, shows clearly the enhancement of the mesopore structure in all biocarriers as compared to the parent 13X zeolite, while Figure 3b, the NLDFT-derived PSD of the desorption branch, shows the multimodal PSD of the biocarriers in the mesopore region.

As regards ZSM-5 and the respective biocarriers, the NLDFT-derived PSD shows the opposite trend (Figure 3c,d). In this case, the parent zeolite is endowed with an extended mesopore structure which is significantly attenuated upon the 3D-printing of the material together with the binder in the form of a log-pile-type biocarrier. 

### 3.2. Density Analysis of 3D-Printed Biocarriers

The addition of a different inorganic binder (clay) to 13-X causes differences in the density of the final material presented in Table 3. The lowest density appears in the mixture of 13X and bentonite. It should be underlined that the material’s low density is desirable in order for the biocarriers to be more easily suspended under agitation conditions (aeration or stirring). The addition of different clays to ZSM-5 zeolite did not cause a significant difference in the densities of the biocarriers. In contrast, zeolite ZSM-5 produced materials with the highest density of 1.97 g/mL of any zeolite/clay combination.

### 3.3. Mechanical Properties Analysis

**Figure 4:**
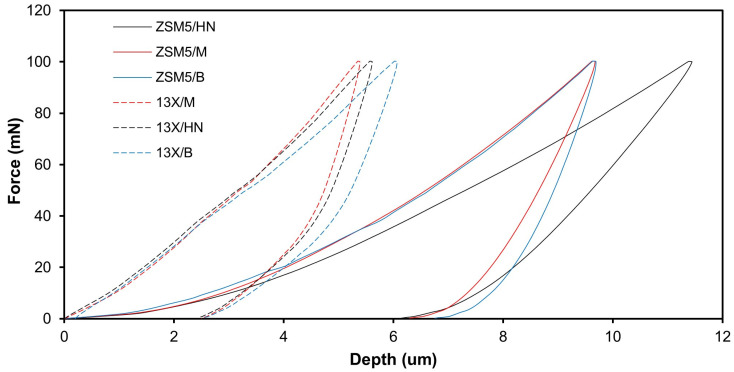
Nanoindentation results of combining different zeolites and binders.

The results obtained from the nanoindentation test data are plotted in Figure 4. The graph shows the relationship between the applied force and the resulting displacement during the indentation test. During the loading curve, a probe is pushed into the surface of the material with continuously increasing force, while the displacement of the probe is recorded, so it is possible to calculate the material’s hardness. Once the maximum force is reached, the probe is then retracted from the material, and the unloading curve is recorded. The unloading curve shows the relationship between the applied force and the displacement as the probe is withdrawn from the material. The material’s modulus of elasticity can be calculated from the slope. The results show that both the hardness and the modulus of elasticity of the biocarriers made of 13X are higher than those of the biocarriers made of ZSM-5. Regarding the effect of the binder on the modulus of elasticity and hardness, there is no clear trend. 

It should also be noted that the hardness and modulus of elasticity are of the same order of magnitude, apart from the modulus of elasticity of the ZSM-5/halloysite nanotubes combination, which is an order of magnitude lower than the rest (Table 4). The greatest hardness was achieved with the 13X/montmorillonite combination with a value of 52.57 MPa. The results obtained from the nanoindentation tests were utilized as input to conduct a finite element (FE) analysis on the mechanical behavior of the biocarriers.

In order to obtain a thorough understanding of the evenness of hardness within the compositions, it is recommended in the literature to provide visual representations such as nanohardness diagrams that depict the interthreads [29,30]. However, in this study, the researchers achieved a high level of uniformity, which is evident from the relatively low values of standard deviations. It is worth noting that all measurements were conducted with an indentation depth of ≈10 μm. These findings validate the previous assumptions proposing that the mechanical properties of the examined materials at extremely small scales, typically in the nanometer range, exhibit significant homogeneity.

**Figure 5:**
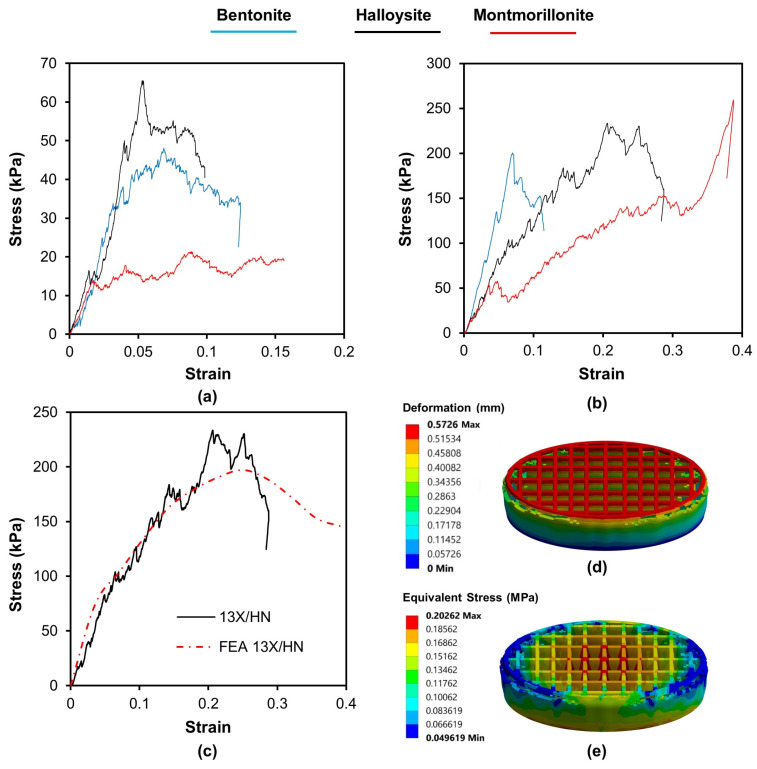
Compression results for (**a**) ZSM-5 zeolite combined with different binders and (**b**) 13X zeolite combined with different binders and (**c**) FEA-generated compression test results compared to experimental values of 13X specimens along with (**d**) vertical deformation and (**e**) stress distribution of the structure under compression load, utilizing the 13X/HΝTs material properties in the FE model.

Compression tests were carried out on samples and representative stress–strain curves are presented in Figure 5. All specimens presented a multipeak profile due to successive noncritical failures. The repetition of this behavior produces a jagged stress–strain curve until a relatively sharp decrease in stress, which indicates the critical failure. The results of the compression tests show that the biocarriers made of 13X present a higher resistance to compression (Table 5). The effect of inorganic binders does not follow any common trend in biocarriers. The highest compressive strength is shown by the combination of 13X and montmorillonite with a value of 261 KPa. Furthermore, to assess the stress response of the 3D-printed specimens under compression, a computational model was utilized. This model was developed through the ANSYS software and incorporated initial stress–strain values obtained from nanoindentation tests of the 3D materials. A vertical velocity was applied in increments to the top plate of each lattice structure, and the resulting reaction force at the bottom was obtained, taking into account a fixed boundary condition. The vertical displacement values were obtained from the experimental results. Deformation was taken into consideration, and the resulting forces were compared to the experimental results. A hexahedral mesh was used for both the top compression plate and the 3D-printed biocarriers. Figure 5c presents the stress–strain behavior obtained from the finite element analysis (FEA) results, which showed a strong correlation between the experimental compression tests and the force-displacement data generated by the FEA simulations for the 3D-printed specimens. However, it is important to note that at larger displacements, the experimental curves began to diverge more from the FEA simulation because the 3D printing defects had a greater impact on the bending response. The material model parameters were analyzed to minimize the difference between the simulated and experimental stress–strain data. As a result, the deformation and the equivalent stress distribution of the 3D-printed lattice structures under compressive load, as presented in Figure 5d,e, could precisely identify the high-stress regions of the structures. Based on the mechanical test results, it can be concluded that the computationally generated (FEA) compression test data, with the assistance of actual measurements, could be a reliable approach to evaluate the mechanical deformation behavior of 3D-printed lattice biocarriers.

### 3.4. Scanning Electron Microscopy (SEM) Analysis of the Novel 3D-Printed Biocarriers

**Figure 6:**
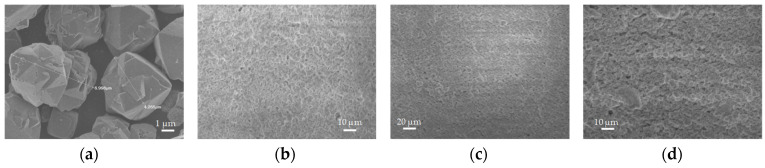
SEM images of (**a**) pristine 13X particles, (**b**) 13X/bentonite, (**c**) 13X/halloysite nanotubes, and (**d**) 13X/montmorillonite 3D-printed biocarriers.

**Figure 7:**
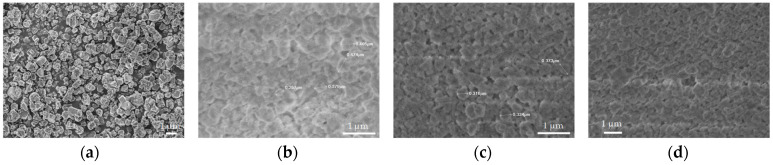
SEM images of (**a**) pristine ZSM-5 particles, (**b**) ZSM-5/bentonite, (**c**) ZSM-5/halloysite nanotubes, and (**d**) ZSM-5/montmorillonite 3D-printed biocarriers.

**Figure 8:**
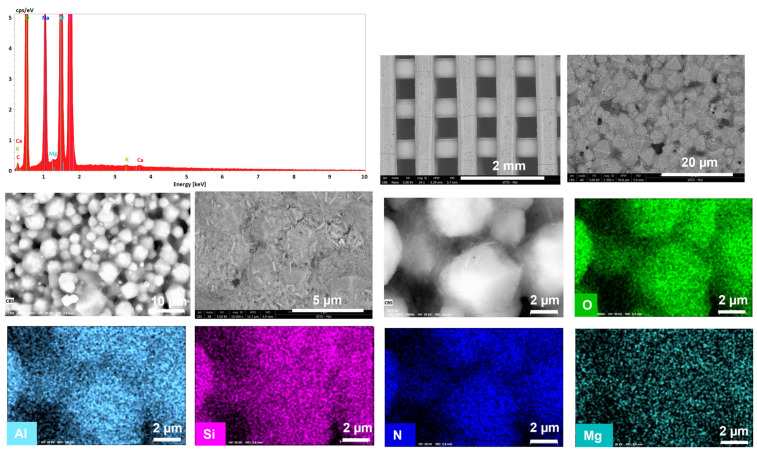
Energy spectrum (top left), SEM images (black and white), and EDX (color) mappings showing macrostructure, microstructure, and composition within the 3D-printed biocarriers, including uniform dispersion of the 13X/halloysite nanotubes fibers in the 13X matrix.

**Figure 9:**
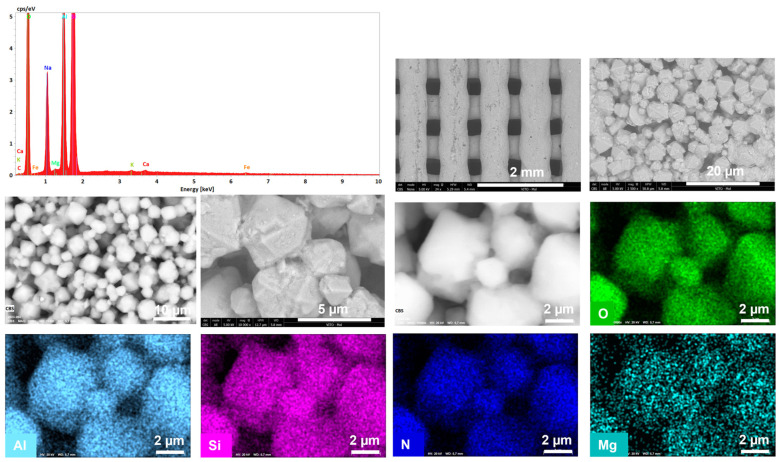
Energy spectrum (top left), SEM images (black and white), and EDX (color) mappings showing macrostructure, microstructure, and composition of the 3D-printed biocarriers, including uniform dispersion of the montmorillonite fillers in the 13X matrix.

**Figure 10:**
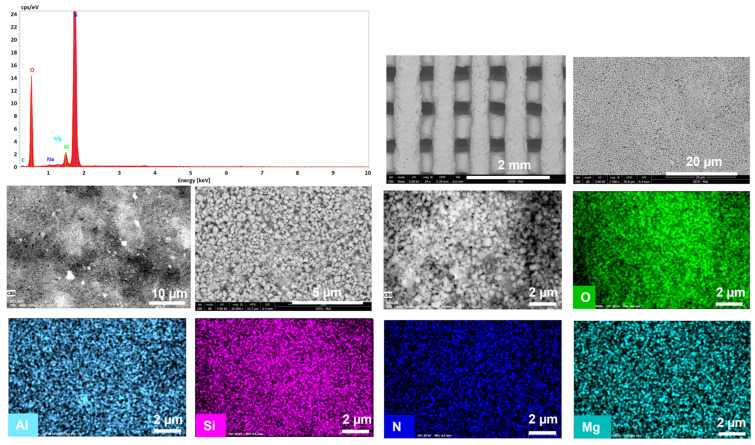
Energy spectrum (top left), SEM images (black and white), and EDX (color) mappings showing macrostructure, microstructure, and composition of the 3D-printed biocarriers, including uniform dispersion of bentonite in the ZSM-5 matrix.

Figure 6a and Figure 7a show a significant difference in particle size of the parental zeolitic materials. In particular, 13X exhibits a narrow crystal size distribution of 4–5 μm and a well-defined morphology, and this possibly explains the synergistic effects achieved in the pore structural features of the respective 3D-printed scaffolds as discussed in Section 3.1. In particular, the larger particle size (5 μm as compared to 0.5 μm for ZSM-5), allows for the existence of more space between the 13X particles. As a result, the particles of the clay binders were better dispersed between the particles of the 13X zeolite leading to higher mechanical stability of the respective biocarriers as well as to the creation of extended mesoporosity. A high degree of dispersion was ascertained by scanning electron microscopy in all three combinations, with the 13X/halloysite nanotubes biocarriers (Figure 6b and Figure 8) exhibiting even more uniform distribution resulting in better mechanical stability (compression behavior). This property of the material is due to the combination of the high specific surface area of the 13X zeolite with the properties of the halloysite nanotubes, due to their nanotubular structure with a diameter of 30–70 nm and a length of 1–3 μm, to better disperse and nest in the void space between the zeolite particles. The 13X/montmorillonite biocarrier also demonstrates comparable behavior (Figure 6d and Figure 9). Similar behavior was also observed in the ZSM-5-zeolite-based biocarriers, with the ZSM-5/bentonite biocarrier (Figure 7b and Figure 10) showing the best dispersion.

### 3.5. X-ray Diffraction (XRD) Analysis of the Novel 3D-Printed Biocarriers

**Figure 11:**
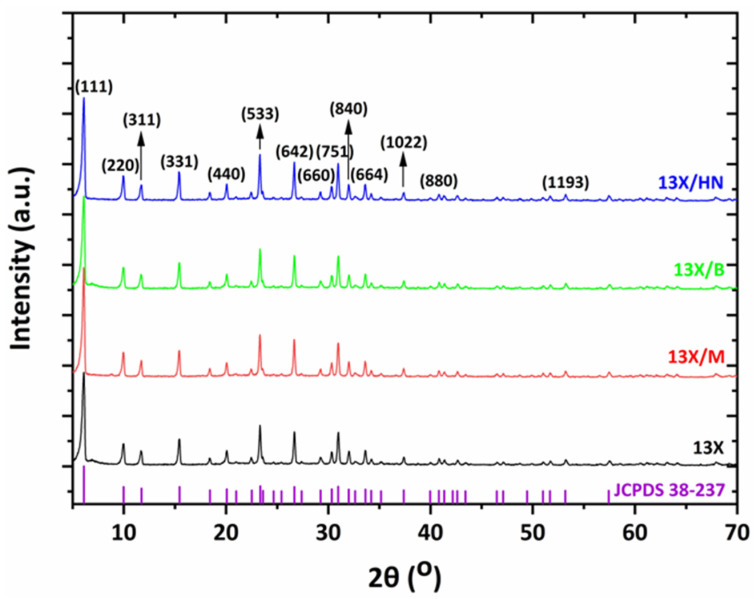
XRD spectra of 13X-based zeolite 3D-printed biocarriers.

**Figure 12:**
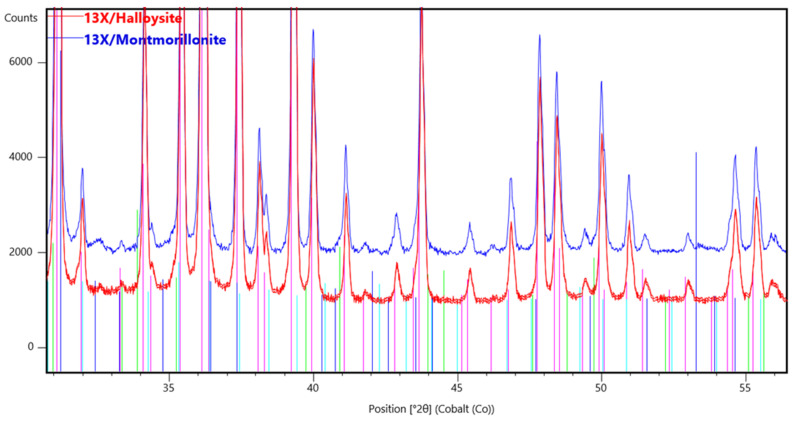

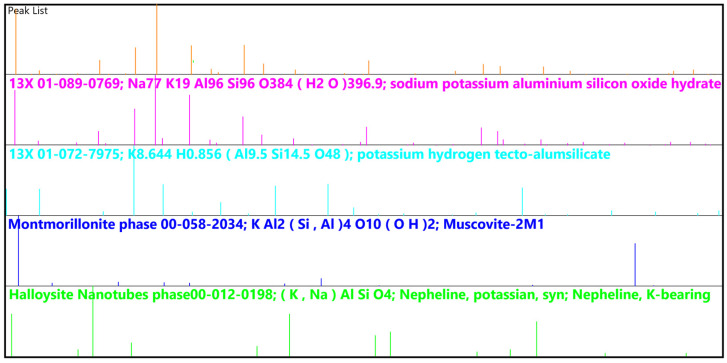
XRD phase analysis for 3D-printed 13X/halloysite nanotubes and 13X/montmorillonite biocarriers (after calcination at 500 °C), showing the main 13X reflections and other phases originating from halloysite nanotubes and montmorillonite.

The XRD spectra of zeolite 13X with all three combinations of inorganic materials (bentonite, halloysite nanotubes, and montmorillonite) show the characteristic peaks of zeolite 13X, which overlap the peaks of the inorganic binders (Figure 11). This is due to the large amount of zeolite 13X in relation to the corresponding inorganic materials, as the percentage of bentonite, halloysite nanotubes, or montmorillonite in the final biocarrier is 11%. This percentage is calculated from the quantities of raw materials used to prepare the mixture. Notwithstanding, the obtained XRD diffraction patterns with the illustrated corresponding planes are consistent with the literature data on typical 13X zeolite structures [31,32,33]. Characteristic peaks are assigned to cubic sodium aluminum silicate hydrate phases (Fd3m space group) [32]. See Figure 12 for more details of the main 13X phase present and other reflections identified in the selected best performing 3D-printed 13X/halloysite nanotubes and 13X/montmorillonite samples.

**Figure 13:**
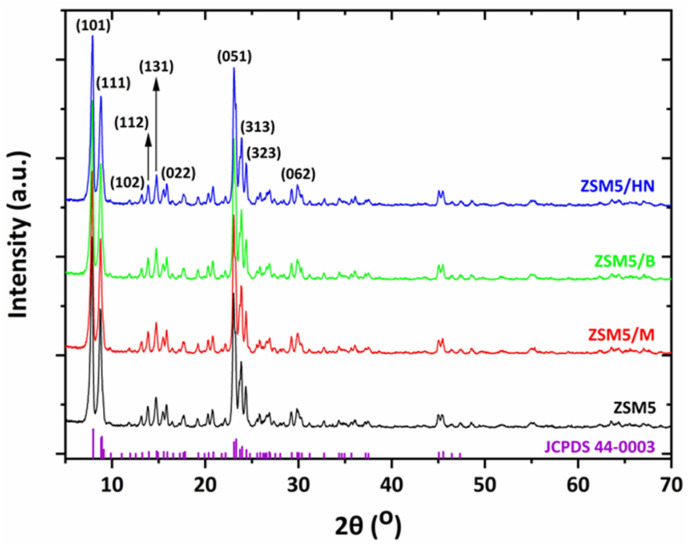
XRD spectra of ZSM-5-based zeolite 3D-printed biocarriers.

**Figure 14:**
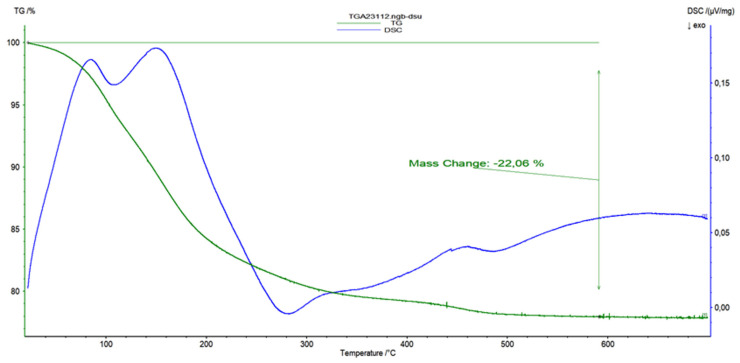
TGA-DTG curves recorded in air as a function of temperature from RT to 700 °C: TGA mass loss curve (green) for 3D-printed 13X/halloysite nanotubes (a weight loss of 22% is associated firstly with the loss of water at up to 200 °C and then methylcellulose loss at 550 °C).

This occurrence is also observed in the XRD spectra of the ZSM-5 zeolite with bentonite, halloysite, and montmorillonite nanotubes, which is due to the final biocarrier’s equally low percentage of inorganic binders (11%) (Figure 13). These biocarriers exhibit the characteristic XRD pattern of the MFI zeolite structure [34,35] in line with the standard JCPDS file (card No. 44-0003) with the diffraction peaks at 2θ = 7.9°, 8.8°, 13.2°, 13.9°, 14.8°, 15.9°, 23.0°, 23.9°, 24.4°, and 29.8° that are associated with (101), (111), (102), (112), (131), (022), (051), (313), (323), and (062) planes, respectively. Additional information could be extracted from TGA-DTG curves for 3D-printed 13X/halloysite nanotubes (Figure 14). Furthermore, in the spectra of ZSM-5/bentonite, ZSM-5/halloysite nanotubes, and ZSM-5/montmorillonite, an attenuation in the intensity of the Bragg reflections between 22.5° and 25° was observed. The decreased peak intensity of the composites resulted from the incorporation of the clays and colloidal silica and the high calcination temperature [34].

## 4. Conclusions

In this work, different aluminosilicate binders were used in order to produce zeolite monoliths through 3D printing. The various zeolite–binder combinations led to complex materials with improved properties and increased performance for a range of key applications.

The quite distinct, innovative pore structural characteristics and crystal size of the parental zeolites, combined with the various morphological features of the involved inorganic binders, produced different types of bonding at the interface boundary between the two basic constituents of the biocarriers. This had a significant impact on the properties of interest such as the pore structure and the mechanical strength. In the case of the 13X zeolite, a quite extended mesopore structure, which was absent in the parental material, was formed in all the 3D-printed formulations. Especially in the case of the 13X/halloysite nanotubes biocarrier, synergistic effects of the pore structural features occurred due to the mixing of the two components. Thus, the specific surface area and total pore volume of the 3D-printed scaffolds significantly increased as compared to the 13X zeolite, while the micropore volume remained unaffected. In addition, the 13X/halloysite nanotubes biocarrier exhibited enhanced mechanical properties. The obtained results show that the crystal size and uniformity of the basic component are the fundamental properties defining to a high extent the quality of the 3D-printed biocarrier. A larger particle size makes more space available for the incorporation of the inorganic binder, leading to robust interfacial binder bridges with the aggregated zeolite particles. Moreover, the unique structure and properties of HNTs are crucial for the reinforcement of the printed zeolite scaffolds. This is attributed to the high aspect ratio, nanotubular structure, and high strength of the introduced HNTs.

Concerning the Archimedes density measurements, the inclusion of various clays in ZSM-5 zeolite did not result in a notable variation in the densities of the biocarriers. The combination of 13X and bentonite exhibited the lowest density. The findings of nanoindentation tests indicate that the biocarriers composed of 13X exhibited a higher level of hardness and modulus of elasticity compared to those made of ZSM-5. It is noteworthy that the hardness and modulus of elasticity are generally within the same range, except for the ZSM-5/halloysite nanotubes combination, which had a significantly lower modulus of elasticity, one order of magnitude below that of the others, while the highest hardness value was attained with the 13X/montmorillonite combination. The compression test results indicate that biocarriers made of 13X exhibited greater resistance to compression. The impact of inorganic binders is not consistent across all biocarriers. The combination of 13X and montmorillonite offered the highest compressive strength. In addition, a computational model was employed to evaluate the stress response of the 3D-printed specimens under compression. The mechanical test results suggest that combining computationally generated (FEA) compression test data with actual measurements provides a dependable method to assess the mechanical deformation behavior of the 3D-printed lattice biocarriers.

## Figures and Tables

**Table 1:** The percentage of zeolite and inorganic binder content.

	Material		Paste Content (%)	Percentage of Zeolites and Clays (%)	Material Quantities (g)
13X/Bentonite	Zeolite	13X	45	89	16
	Inorganic Binder	Bentonite	6	11	2
	Alumina Silicate Binder	Ludox AS-40	14		5
	Moisture	Water	34		17.2
	Organic Binder	Methylcellulose	1		0.3
	Total amount of paste	40.5 g			
	Total Solids	50%			
13X/Halloysite Nanotubes	Zeolite	13X	50	89	22.4
	Inorganic Binder	Halloysite Nanotubes	6	11	2.8
	Alumina Silicate Binder	Ludox AS-40	16		7
	Moisture	Water	27		13.7
	Organic Binder	Methylcellulose	1		0.3
	Total amount of paste	46.2 g			
	Total Solids	61%			
13X/Montmorillonite	Zeolite	13X	49	89	16
	Inorganic Binder	Montmorillonite	6	11	2
	Alumina Silicate Binder	Ludox AS-40	15		5
	Moisture	Water	29		12.2
	Organic Binder	Methylcellulose	1		0.3
	Total amount of paste	35.4 g			
	Total Solids	57%			
ZSM-5/Bentonite	Zeolite	ZSM-5	37	89	8
	Inorganic Binder	Bentonite	5	11	1
	Alumina Silicate Binder	Ludox AS-40	12		2.5
	Moisture	Water	45		11.7
	Organic Binder	Methylcellulose	2		0.4
	Total amount of paste	23.6 g			
	Total Solids	44%			
ZSM-5/Montmorillonite	Zeolite	ZSM-5	43	89	8
	Inorganic Binder	Montmorillonite	5	11	1
	Alumina Silicate Binder	Ludox AS-40	14		2.5
	Moisture	Water	37		7.8
	Organic Binder	Methylcellulose	1		0.2
	Total amount of paste	19.5 g			
	Total Solids	52%			
ZSM-5/Halloysite Nanotubes	Zeolite	ZSM-5	43	89	8
	Inorganic Binder	Montmorillonite	5	11	1
	Alumina Silicate Binder	Ludox AS-40	14		2.5
	Moisture	Water	37		6.8
	Organic Binder	Methylcellulose	1		0.2
	Total amount of paste	18.5 g			
	Total Solids	55%			

**Table 2:** Surface area, pore volume, and size of innovative 3D-printed biocarriers compared to original materials.

	Specific Surface Area (m^2^/g)	Total Pore Volume (cc/g)	Micropore Volume (cc/g)	Mesopore Volume (cc/g)	Micropore Diameter (Å)	Mesopore Diameter (Å)
13X	688	0.35	0.29	0.06	10.2	55
ZSM-5	549	0.57	0.21	0.36	5	42
Bentonite	52	0.15	0.02	0.13	na	55
Montmorillonite	32	0.16	0.01	0.15	na	55
Halloysite Nanotubes	89	0.32	0.03	0.29	na	55 and 116
13X/Bentonite	590	0.42	0.24	0.18	9	42, 116
13X/Halloysite Nanotubes	711	0.49	0.29	0.20	9	34, 55, 97
13X/Montmorillonite	687	0.42	0.28	0.14	9	39, 54, 94
ZSM-5/Bentonite	527	0.56	0.21	0.35	9.4	35, 51, 116
ZSM-5/Montmorillonite	397	0.32	0.16	0.16	9	24, 35, 54
ZSM-5/Halloysite Nanotubes	383	0.37	0.15	0.22	9	35, 53, 78

**Table 3:** Density measurements of 3D-printed biocarriers.

3D-Printed Biocarriers	Density of Biocarriers (g/cm^3^)
13X	0.60
ZSM-5	0.4–0.8
Bentonite	0.8–1.0
Montmorillonite	0.52
Halloysite Nanotubes	2.53
13X/Bentonite	1.42 ± 0.03
13X/Montmorillonite	1.64 ± 0.03
13X/Halloysite Nanotubes	1.67 ± 0.02
ZSM-5/Bentonite	1.97 ± 0.01
ZSM-5/Montmorillonite	1.98 ± 0.16
ZSM-5/Halloysite Nanotubes	1.94 ± 0.20

**Table 4:** Hardness and elastic modulus values obtained through nanoindentation.

3D-Printed Biocarriers	Hardness (MPa)	Elastic Modulus (MPa)
13X/Bentonite	44.35 ± 5.39	3378.67 ± 383.89
13X/Halloysite Nanotubes	45.64 ± 5.47	3422 ± 130.11
13X/Montmorillonite	52.57 ± 4.60	3223 ± 335.71
ZSM-5/Bentonite	38.45 ± 7.64	1347.75 ± 47.53
ZSM-5/Montmorillonite	38.21 ± 2.37	1188.5 ± 204.19
ZSM-5/Halloysite Nanotubes	29.01 ± 6.01	545.5 ± 52.39

**Table 5:** Compression test results.

3D-Printed Biocarriers	Compressive Strength (kPa)
13X/Bentonite	175.85 ± 34.93
13X/Halloysite Nanotubes	215.89 ± 35.36
13X/Montmorillonite	261 ± 2.28
ZSM-5/Bentonite	50.95 ± 10.38
ZSM-5/Montmorillonite	20.52 ± 4.90
ZSM-5/Halloysite Nanotubes	57.78 ± 11.05

## Data Availability

The data presented in this study are available upon request from the corresponding author.
